# Traveling the Path to Stroke Caregiver Readiness

**DOI:** 10.1093/geroni/igaa057.819

**Published:** 2020-12-16

**Authors:** Barbara Lutz, Michelle Camicia

**Affiliations:** 1 University of North Carolina-Wilmington, Wilmington, North Carolina, United States; 2 Kaiser Foundation Rehabilitation Center, Vallejo, California, United States

## Abstract

Family members are often poorly prepared to assume the caregiving role post-stroke leaving them feeling overwhelmed, frustrated, and abandoned by the healthcare system leading to physical, mental, and emotional strain. To address this, we developed and tested the Preparedness Assessment for the Transition Home after stroke (PATH-s) instrument based on a theoretical framework for improving stroke caregiver readiness. Consecutive studies were conducted over the past 10 years to 1) develop the caregiver readiness theoretical model identifying gaps in caregiver preparation in 80 interviews with caregivers and stroke survivors as they transitioned home from inpatient rehabilitation care; 2) develop and validate the PATH-s instrument with 183 caregiver-stroke survivor dyads, and 3) develop and implement a corresponding catalogue of interventions developed in consultation with 5 expert rehabilitation nurse case managers to improve stroke caregiver readiness. The Improving Caregiver Readiness Model has 2 preparedness domains; commitment and capacity and six sub-domains. In a factor analysis each domain/sub-domain subscale in the PATH-s demonstrated satisfactory internal consistency (a=0.69-0.86). The overall mean score was 3.11 (range 1.68 to 4.00) with high internal consistency reliability (a=0.90). The PATH-s is highly correlated with the Preparedness for Caregiving Scale. The stroke survivor’s total FIM score at discharge had a small but significant correlation with the PATH-s. Case managers find the PATH-s results and corresponding interventions helpful in tailoring transitional care plans. Caregivers worldwide describe the negative impacts of providing stroke care post-discharge. The Path to Stroke Caregiver Readiness Program shows promise for improving stroke caregiver preparation for discharge home.

